# Fragmented mitochondrial genomes of the rat lice, *Polyplax asiatica* and *Polyplax spinulosa*: intra-genus variation in fragmentation pattern and a possible link between the extent of fragmentation and the length of life cycle

**DOI:** 10.1186/1471-2164-15-44

**Published:** 2014-01-18

**Authors:** Wen-Ge Dong, Simon Song, Dao-Chao Jin, Xian-Guo Guo, Renfu Shao

**Affiliations:** 1 Institute of Entomology, Guizhou University, and the Provincial Key Laboratory for Agricultural Pest Management in Mountainous Region, Guiyang 550025, China; 2 Institute of Pathogens and Vectors, Dali University, and the Key Laboratory for Preventing and Controlling Plague in Yunnan Province, Dali 671000, China; 3 GeneCology Research Centre, Faculty of Science, Health, Education and Engineering, University of the Sunshine Coast, Maroochydore, Queensland 4556, Australia

**Keywords:** Mitochondrial genome, Genome fragmentation, Minichromosome, Chromosome evolution, Sucking lice

## Abstract

**Background:**

Blood-sucking lice (suborder Anoplura) parasitize eutherian mammals with 67% of the 540 described species found on rodents. The five species of blood-sucking lice that infest humans and pigs have fragmented mitochondrial genomes and differ substantially in the extent of fragmentation. To understand whether, or not, any life-history factors are linked to such variation, we sequenced the mt genomes of *Polyplax asiatica* and *Polyplax spinulosa*, collected from the greater bandicoot rat, *Bandicota indica*, and the Asian house rat, *Rattus tanezumi*, respectively.

**Results:**

We identified all of the 37 mitochondrial genes common to animals in *Polyplax asiatica* and *Polyplax spinulosa*. The mitochondrial genes of these two rat lice are on 11 circular minichromosomes; each minichromosome is 2–4 kb long and has 2–7 genes. The two rat lice share the same pattern for the distribution of the protein-coding genes and ribosomal RNA genes over the minichromosomes, but differ in the pattern for the distribution of 8 of the 22 transfer RNA genes. The mitochondrial genomes of the *Polyplax* rat lice have 3.4 genes, on average, on each minichromosome and, thus, are less fragmented than those of the human lice (2.1 and 2.4 genes per minichromosome), but are more fragmented than those of the pig lice (4.1 genes per minichromosome).

**Conclusions:**

Our results revealed distinct patterns of mitochondrial genome fragmentation within the genus *Polyplax* and, furthermore, indicated a possible inverse link between the extent of mitochondrial genome fragmentation and the length of life cycle of the blood-sucking lice.

## Background

Lice in the suborder Anoplura are wingless, exclusive blood-sucking insects and are permanent ectoparasites of eutherian mammals [1,2]. Blood-sucking lice evolved from chewing lice ~100 million years ago (Mya) and diversified rapidly ~65 Mya with their mammalian hosts [3]. More than 540 species of blood-sucking lice have been described and are classified into 15 families and 50 genera [4-7]. Twelve of the 29 recognized mammalian orders and ~840 mammalian species are hosts of blood-sucking lice. The diversity of blood-sucking lice mirrors that of their mammalian hosts with 67% of the described species found on rodents [2,5,7]. Each species of mammalian host is usually parasitized by a single species of blood-sucking lice, but there are exceptions as many mammalian species are hosts of multiple species of blood-sucking lice (up to seven species) [7]. Vice versa, each species of blood-sucking lice usually parasitizes only one species of mammals, but many species of blood-sucking lice parasitize multiple species of mammals that are closely related phylogenetically (up to 39 species) [6].

Adding to their unique, exclusive blood-feeding life style and the high host-specificity (relative to other ectoparasites), recent studies revealed that blood-sucking lice have an unusual, fragmented mitochondrial (mt) genome organization. For insects and other bilateral animals, the mt genome is usually a single, circular chromosome, 13–20 kb long, with 37 genes: 13 for proteins, two for ribosomal RNAs and 22 for transfer RNAs [8,9]. The mt genomes of the human body louse, *Pediculus humanus*, and the human head louse, *Pediculus capitis*, however, have 20 minichromosomes; each minichromosome has 1–3 genes and is 3–4 kb in size [10,11]. The 34 mt genes identified in the human pubic louse, *Pthirus pubis*, are on 14 minichromosomes; each minichromosome has 1–5 genes and is 1.8–2.7 kb in size [10,11]. The domestic pig louse, *Haematopinus suis*, and the wild pig louse, *Haematopinus apri*, have their 37 mt genes on nine minichromosomes; each minichromosome has 2–8 genes and is 3–4 kb in size [12].

The fragmented mitochondrial genomes of the human lice and the pig lice represent the most radical departure to date in bilateral animals from the typical, single-chromosome organization of mt genomes, although multipartite mt genomes have also been observed in the rotifer, *Brachionus plicatilis*, and the booklouse, *Liposcelis bostrychophila*, which have two mt chromosomes [13,14], and the potato cyst nematode, *Globodera pallida*, which has six chromosomes [15]. Outside bilateral animals, highly fragmented mt genomes were observed in ichthyosporean protists [16], diplonemid protists [17] and box jellyfish [18].

Although all having fragmented mt genomes, the human lice and the pig lice differ substantially in the extent of mt genome fragmentation and in the distribution of mt genes over the minichromosomes [12]. To understand whether, or not, any life-history factors are linked to such variation, we sequenced the mt genomes of two species of blood-sucking lice that parasitize rats, *Polyplax asiatica* (Ferris 1923) and *Polyplax spinulosa* (Burmeister 1839). *Polyplax* is one of the most species-rich genus of the suborder Anoplura with 78 described species, second only to the genus *Hoplopleura*, which has 141 described species [6]. *Polyplax* species shared their most recent common ancestor (MRCA) with primate lice ~47 Mya and their MRCA with pig lice ~67 Mya [3]. *Po. asiatica* infests the greater bandicoot rat, *Bandicota indica*, three other related species of rats and occasionally the Asian house shrew, *Suncus murinus*[7,19]. *Po. spinulosa* infests the Asian house rat, *Rattus tanezumi*, the brown rat, *Rattus norvegicus*, the black rat, *Rattus rattus*, and six other related species of rats [6,7]. While *Po. asiatica* is only found in Asia, *Po. spinulosa* has a worldwide distribution and is responsible, as a vector, for the transmission of pathogenic microorganisms such as *Mycoplasma haemomuris* (formerly known as *Haemobartonella muris*), *Rickettsia typhi*, *Trypanosoma lewisi*, *Borrellia duttoni* and *Brucella brucei*[20-22]. We show that both *Po. asiatica* and *Po. spinulosa* have fragmented mt genomes; these two species, however, differ in the pattern of mt genome fragmentation. Furthermore, our comparison among the rat lice, the human lice and the pig lice indicates a possible inverse link between the extent of mt genome fragmentation and the length of life cycle of these lice.

## Methods

### Collection of rats and lice

The blood-sucking lice, *Po. asiatica* and *Po. spinulosa*, were collected in 2011 from the greater bandicoot rat, *B. indica*, and the Asian house rat, *R. tanezumi*, respectively, in Jinping county, Yunnan province, China. The rats were caught with trap-cages set indoors (farmers’ houses, barns and stables) and outdoors (farmlands, scrublands and woodlands). Alive rats trapped were placed individually in pre-marked cotton bags and transferred to laboratory for species identification and parasitological check. Blood-sucking lice on the body surface of each rodent host were collected and preserved in 95% ethanol at −20°C prior to DNA extraction. Samples of *Po. asiatica* and *Po. spinulosa* and their rat hosts were deposited in the Institute of Pathogens and Vectors, Dali University. The capture of rodents was approved by health authorities in Yunnan province, China. Animal protocols and procedures were approved by the animal ethics committees at Guizhou University and Dali University.

### DNA extraction, mitochondrial genome amplification and sequencing

**Figure 1:**
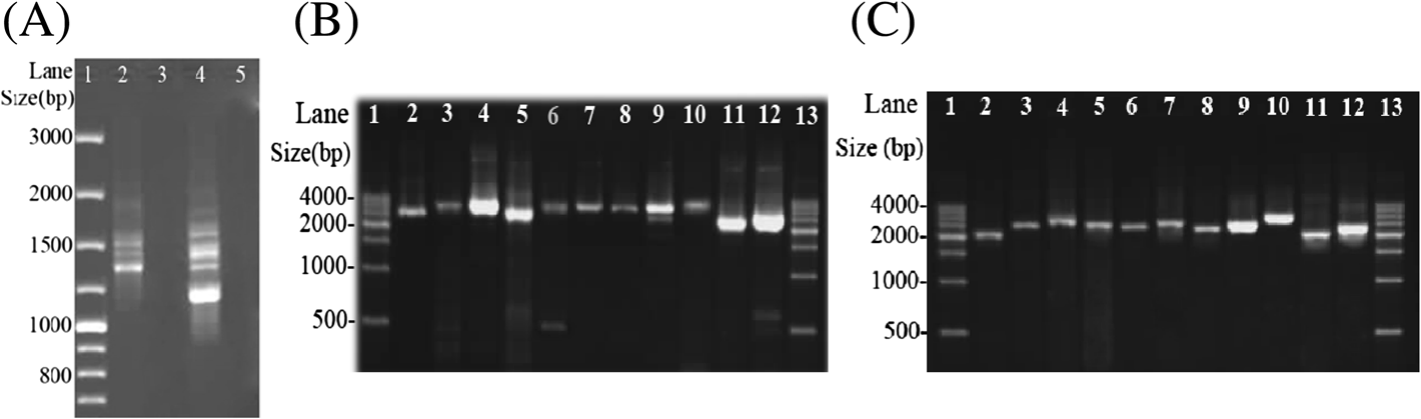
**PCR amplification of the mitochondrial (mt) minichromosomes of the *Polyplax *rat lice. (A)** Lane 1: GeneRuler®100 bp DNA Ladder (Thermo Scientific). Lane 2: PCR amplicons generated with primer pair 57F-57R that spans the coding region of each mitochondrial (mt) minichromosome of *Polyplax asiatica*. Lane 4: PCR amplicons generated with primer pair 301F-301R that spans the coding region of each mt minichromosome of *Polyplax spinulosa*. **(B)** PCR verification of the mt minichromosomes of *Po. asiatica*. Lane 1 and 13: 500 bp DNA Ladder (TIANGEN). Lane 2–12: PCR amplicons from the 11 minichromosomes of *Po. asiatica*: *atp8*-***atp6***, *trnE*-***cob***-*trnI*, ***cox1***-*trnL*_*2*_(*taa*), *trnD*-*trnY*-***cox2***-*nad6*, ***trnR***-*nad4L*-*cox3*-*trnA*, ***trnS***_***1***_(***tct***)-*trnS*_*2*_(*tga*)-*nad1*-*trnT*-*trnG*-*nad3*-*trnW*, *trnQ*-***nad2***-*trnN*-*trnP*, *trnK*-***nad4***-*trnF*, *trnH*-***nad5***, ***rrnS***-*trnC*, *trnM*-*trnL*_*1*_(*tag*)-***rrnL***-*trnV*. **(C)** PCR verification of the mt minichromosomes of *Po. spinulosa*. Lane 1 and 13: 500 bp DNA Ladder (TIANGEN). Lane 2–12: PCR amplicons from the 11 minichromosomes of *Po. spinulosa*: *atp8*-***atp6***, *trnE*-***cob***-*trnI*, ***cox1***-*trnL*_*1*_(*tag*), *trnT*-*trnD*-*trnY*-***cox2***-*nad6*-*trnA*, ***trnR***-*nad4L*-*trnP*-*cox3*, *nad1*-***trnG***-*nad3*-*trnW*, *trnQ*-***nad2***-*trnN*, *trnK*-***nad4***, *trnH*-***nad5***-*trnF*, *trnS*_*1*_ (*tct*)-*trnS*_*2*_ (*tga*)-***rrnS***-*trnC*, *trnM*-*trnL*_*2*_(*taa*)-***rrnL***-*trnV*. Genes from which PCR primers were designed are in bold.

Genomic DNA was extracted from individual louse specimens with DNeasy Tissue kit (QIAGEN). For *Po. asiatica* (sample #57) collected from the greater bandicoot rats, a 532-bp fragment of *cox1* and a 360-bp fragment of *rrnL* were initially amplified by PCR with primer pairs mtd6-mtd9 and16SF-Lx16SR (see Additional file 1). These two pairs of primers target conserved sequence motifs in *cox1* and *rrnL*; the amplicons were sequenced directly using Sanger method at the Tiangen Biotech, Beijing (TBB). Two pairs of specific primers for *Po. asiatica*, cox57F-cox57R and 16S57F-16S57R, were designed from sequences of the *cox1* and *rrnL* fragments. The two specific primers in each pair go outbound with 131 bp and 1 bp, respectively, in between. PCRs with these specific primers amplified two near full-length mt minichromosomes of *Po. asiatica* that contain *cox1* and *rrnL* respectively; these amplicons (3.2 kb and 2.6 kb in size) were sequenced using Sanger method at the TBB. Another pair of primers specific to *Po. asiatica*, 57F-57R, was designed from conserved non-coding sequences that flank the coding regions of the two minichromosomes above. The PCR with 57F-57R primer pair produced a mixture of amplicons ranging from 1 to 2 kb in size, expected from the coding regions of all mt minichromosomes of *Po. asiatica* (Figure 1A). These amplicons were sequenced with Illumina Hiseq 2000 platform at the Beijing Genome Institute, Hong Kong (BGI-HK). The PCR strategy used in this study was developed from the observations we made in previous studies on the human lice and the pig lice that each mt minichromosome has a distinct coding region but a well-conserved non-coding region [10-12].

For *Po. spinulosa* (sample #301) collected from the Asian house rats, a 452-bp fragment of *rrnS* and a 360-bp fragment of *rrnL* were amplified initially by PCR with primer pairs 12SA-12SB and 16SF-Lx16SR (see Additional file 1). These two pairs of primers target conserved sequence motifs in *rrnS* and *rrnL* and the PCR amplicons were sequenced directly using Sanger method at the TBB. Two pairs of specific primers for *Po. spinulosa*, 12S301F-12S301R and 16S301F-16S301R, were designed from sequences of the *rrnS* and *rrnL* fragments. The two specific primers in each pair go outbound and are 1 bp and 89 bp respectively from each other. PCRs with these two pairs of specific primers amplified two mt minichromosomes of *Po. spinulosa* that contain *rrnS* and *rrnL* respectively; these amplicons (2 kb and 2.3 kb in size) were sequenced using Sanger method at the TBB. Another pair of primers specific to *Po. spinulosa*, 301F-301R, was designed from conserved non-coding sequences that flank the coding regions of the two minichromosomes above. The PCR with primer pair 301F-301R produced a mixture of amplicons ranging from 1 to 2 kb in size, expected from the coding regions of all mt minichromosomes of *Po. spinulosa* (Figure 1A). These amplicons were sequenced with Illumina Hiseq 2000 platform at the BGI-HK.

*Taq* DNA Polymerase (Tiangen Biotech) was used in the initial short PCRs with the following cycling conditions: 94°C for 1 min; 35 cycles of 98°C for 10 sec, 45°C for 30 sec, 72°C for 1 min; and a final extension of 72°C for 2 min. LA *Taq* (TaKaRa) was used in the long PCRs with the cycling conditions: 94°C for 1 min, 35 cycles of 98°C for 10 sec, 60–65°C (depending on primers) for 30–40 sec, 68°C for 3 min; and 72°C for 6 min. Positive and negative controls were executed with each PCR. PCR amplicons were checked by agarose gel (1%) electrophoresis; the sizes of PCR amplicons were estimated by comparing with molecular markers. PCR products were purified with Wizard SV Gel and PCR clean-up system (Promega).

### Assembly of Illumina sequence-reads, gene identification and verification of individual mitochondrial minichromosomes

Illumina sequence-reads obtained from the mt minichromosomes of *Po. asiatica* and *Po. spinulosa* were assembled into contigs with Geneious 6.1.6 [23]; the parameters for assembly were minimum overlap identity 98% and minimum overlap 50 bp. tRNA genes were identified using tRNAscan-SE [24] and ARWEN [25]. Protein-coding genes and rRNA genes were identified with BLAST searches of GenBank [26,27]. Identical sequences shared between genes were identified with Wordmatch [28]. Sequence alignments were with Clustal X [29]. The size and circular organization of each mt minichromosome of *Po. asiatica* and *Po. spinulosa* identified by sequence-read assembly were verified by PCR (Figure 1B, 1C). A pair of outbound primers (forward and reverse) was designed from the coding region of each minichromosome (see Additional file 2). The two primers in each pair were next to each other with a small gap or no gap in between. PCRs with these primers amplify the full or near full length of each minichromosome if it has a circular organization. PCR set-up, cycling conditions, agarose gel electrophoresis and size measurement were the same as described above. The nucleotide sequences of the mt genomes of *Po. asiatica* and *Po. spinulosa* have been deposited in GenBank under accession numbers KF647751–KF647772.

## Results

### Mitochondrial genome of *Polyplax asiatica*, the louse of the greater bandicoot rats

**Figure 2:**
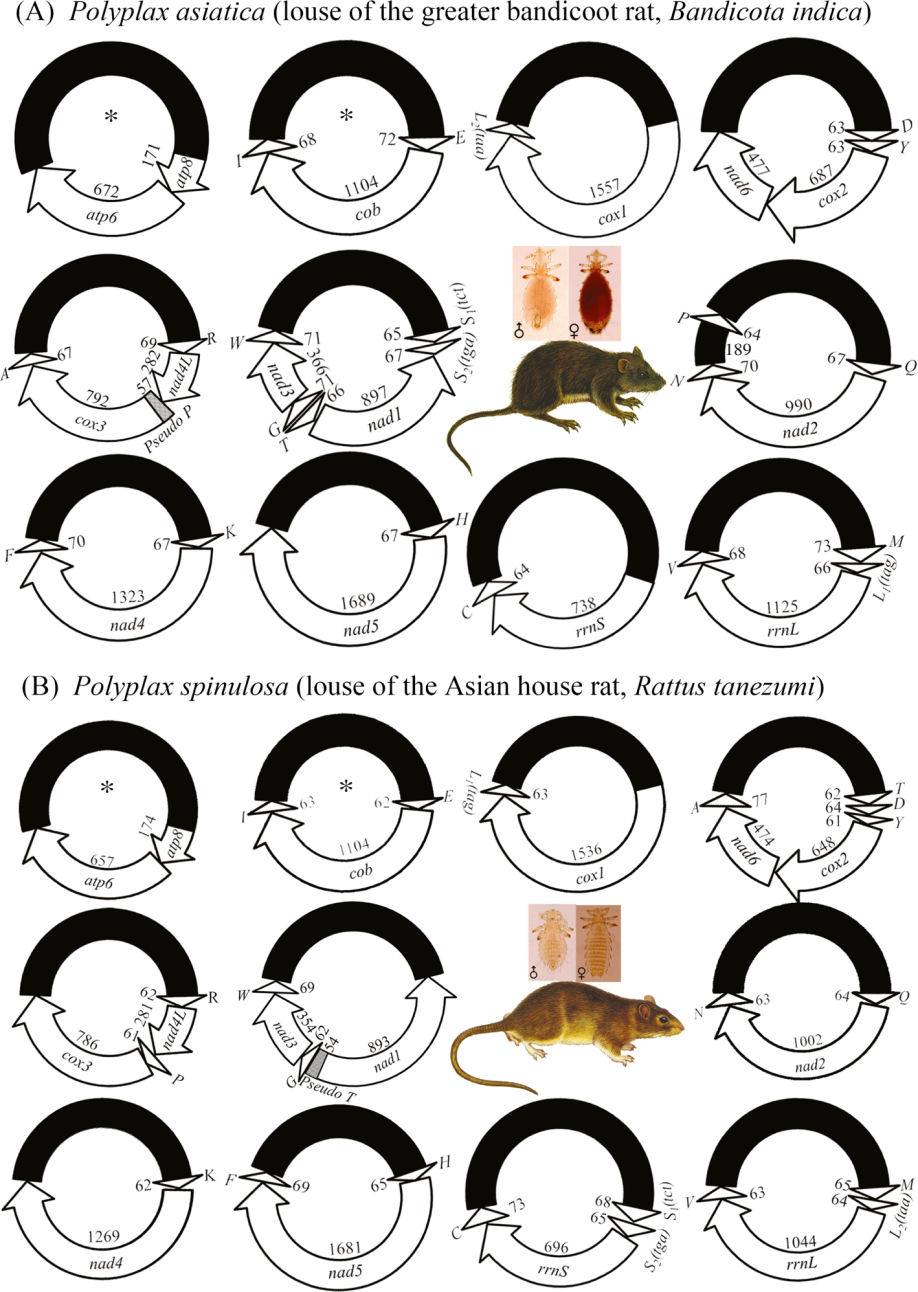
**The mitochondrial** (**mt**) **genomes of *Polyplax asiatica *(A) and *Polyplax spinulosa *(B).** Each minichromosome has a coding region (with gene name, transcription orientation and length indicated) and a non-coding region (in black). Minichromosomes are in alphabetical order by the names of their protein coding and rRNA genes. Abbreviations of gene names are: *atp6* and *atp8* (for ATP synthase subunits 6 and 8), *cox1*–*3* (for cytochrome *c* oxidase subunits 1–3), *cob* (for cytochrome b), *nad1*–*4* and *nad4L* (for NADH dehydrogenase subunits 1–6 and *4 L*), *rrnS* and *rrnL* (for small and large subunits of ribosomal RNA). *tRNA* genes are shown with the single-letter abbreviations of their corresponding amino acids. Minichromosomes that have identical gene content and gene arrangement between the two *Polyplax* species are indicated with asterisk symbols “*”.

We obtained 546,066 sequence-reads from the mt genome of *Po. asiatica* by Illumina sequencing (Table 1). The sequence-reads are 90 bp each; assembly of these sequence-reads into contigs allowed us to identify all of the 37 mt genes typical of animals in *Po. asiatica* distributed over 11 minichromosomes (Figure 2A; Table 1). Each minichromosome is circular, 2.4–3.3 kb in size (Figure 1B), and consists of a coding region and a non-coding region. Each coding region contains two to seven genes, and varies in size from 802 bp for *rrnS*-*trnC* minichromosome to 1,756 bp for *trnH*-*nad5* minichromosome (Table 1; Figure 2A) (Note: minichromosomes are named after their genes hereafter). Seven of the 11 minichromosomes of *Po. asiatica* have one protein-coding or rRNA gene each; the other four minichromosomes have two protein-coding genes each. The 22 tRNA genes are scattered over 10 of the 11 minichromosomes; each minichromosome has one to five tRNA genes except *atp8*-*atp6*, which has no tRNA genes (Figure 2A). Each of the 37 mt genes identified in *Po. asiatica* is present on only one minichromosome; there is no overlap in gene content between different minichromosomes. All of the 37 mt genes have the same orientation of transcription relative to the non-coding region, except *trnT* and *nad1*, which have the opposite orientation of transcription relative to other genes (Figure 2A).

**Table 1:** **Mitochondrial minichromosomes of *Polyplax asiatica *and *Polyplax spinulosa *identified by Illumina sequencing**

**Minichromosome**	**Size of coding region (bp)**	**Number of Illumina sequence-reads**
*atp8*-*atp6* (*atp8*-*atp6*)	838 (832)	73276 (143560)
*E*-*cob*-*I* (*E*-*cob*-*I*)	1242 (1228)	47008 (50108)
*cox1*-*L*_*2*_ (*cox1*-*L*_*1*_ )	1626 (1599)	49653 (49675)
*D*-*Y*-*cox2*-*nad6* (*T*-*D*-*Y*-*cox2*-*nad6*-*A*)	1292 (1399)	35911 (41691)
*R*-*nad4L*-*cox3*-*A* (*R*-*nad4L*-*P*-*cox3*)	1268 (1190)	36763 (64203)
*S*_*1*_-*S*_*2*_-*nad1*-*T*-*G*-*nad3*-*W* (*nad1*-*G*-*nad3*-*W*)	1602 (1419)	21705 (66380)
*Q*-*nad2*-*N*-*P* (*Q*-*nad2*-*N*)	1385 (1134)	24989 (46412)
*K*-*nad4*-*F* (*trnK*-*nad4*)	1465 (1331)	38589 (78061)
*H*-*nad5* (*trnH*-*nad5*-*trnF*)	1756 (1816)	39554 (44417)
*rrnS*-*C* (*S*_*1*_ -*S*_*2*_ -*rrnS* -*C*)	802 (904)	121142 (165740)
*M*-*L*_*1*_-*rrnL*-*V* (*M*-*L*_*2*_-*rrnL*-*V*)	1333 (1237)	57476 (96783)
Total	14609 (14089)	546066 (847030)

Note: Gene arrangements and numbers outside brackets are for *Polyplax asiatica* and those in brackets are for *Polyplax spinulosa*.

**Figure 3:**
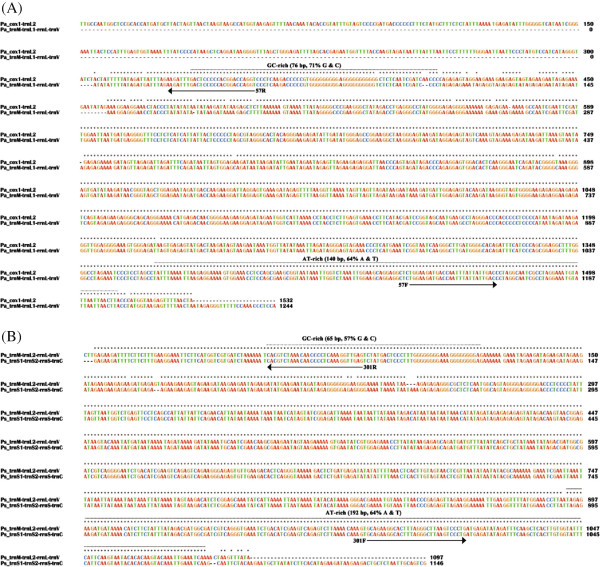
**Alignment of nucleotide sequences in the non-coding regions of the mitochondrial minichromosomes of *Polyplax asiatica *(A) and *Polyplax spinulosa *(B).** 57F and 57R are the primers used to amplify the coding regions of all mitochondrial minichromosomes of *Po. asiatica*. 301F and 301R are the primers used to amplify the coding regions of all mitochondrial minichromosomes of *Po. spinulosa*.

We sequenced the full-length non-coding regions, 1,532 bp and 1,244 bp respectively, of two mt minichromosomes of *Po. asiatica*: *cox1*-*trnL*_*2*_ and *trnM*-*trnL*_*1*_-*rrnL*-*trnV*. The length variation between the two non-coding regions is due to a 304-bp section that is present only in *cox1*-*trnL*_*2*_ minichromosome lying downstream the coding region and a 25-bp section that is present only in *trnM*-*trnL*_*1*_-*rrnL*-*trnV* minichromosome lying upstream the coding region (Figure 3A). Excluding these two sections, the non-coding regions of the two minichromosomes have 96% identity to each other. As in the human lice and the pig lice [11,12], an AT-rich motif (140 bp, 64% A and T) is present in the non-coding regions of *Po. asiatica* upstream the 5’-end of the coding region, whereas a GC-rich motif (76 bp, 71% G and C) is present downstream the 3’-end of the coding region (Figure 3A). In addition to the full-length non-coding region sequences of the two minichromosomes, we also obtained partial sequences of the non-coding regions, 101–169 bp and 49–449 bp respectively, upstream and downstream the coding regions of the other nine minichromosomes of *Po. asiatica*. Two highly conserved sequence-motifs, 101 bp and 44 bp long respectively, are present in the sections of the non-coding regions upstream and downstream the coding regions of all of the 11 minichromosomes (see Additional file 3).

### Mitochondrial genome of *Polyplax spinulosa*, the louse of the Asian house rats

We obtained 847,030 sequence-reads from the mt genome of *Po. spinulosa* by Illumina sequencing (Table 1). As above for *Po. asiatica*, these sequence-reads are 90 bp each in length. We assembled these sequence-reads into contigs and identified all of the 37 mt genes typical of animals in *Po. spinulosa*. These genes are on 11 minichromosomes; each minichromosome is 2.0–2.9 kb in size and has a circular organization (Figure 1C; Figure 2B). As in *Po. asiatica*, seven of the 11 minichromosomes of *Po. spinulosa* have one protein- or rRNA-coding gene each; the other four minichromosomes have two protein-coding genes each. The 22 tRNA genes are on 10 of the 11 minichromosomes; each minichromosome has one to four tRNA genes except *atp8*-*atp6*, which has no tRNA genes (Figure 2B). Each minichromosome has a coding region and a non-coding region. The coding region of each minichromosome contains two to six genes, and varies in size from 832 bp for *atp8*-*atp6* minichromosome to 1,816 bp for *trnH*-*nad5*-*trnF* minichromosome. With the only exception of *nad1*, all of the mt genes of *Po. spinulosa* have the same orientation of transcription relative to the non-coding regions (Figure 2B).

We sequenced the full-length non-coding regions, 1,097 bp and 1,146 bp respectively, of two mt minichromosomes of *Po. spinulosa*: *trnM*-*trnL*_*2*_-*rrnL*-*trnV* and *trnS*_*1*_-*trnS*_*2*_-*rrnS*-*trnC*. The length variation between the two non-coding regions is due to a 53-bp section that is present only in *trnS*_*1*_-*trnS*_*2*_-*rrnS*-*trnC* minichromosome lying upstream the coding region (Figure 3B). Excluding the 53-bp section, the non-coding regions of the two minichromosomes have 98% identity to each other. As above in *Po. asiatica*, an AT-rich motif (192 bp, 64% A and T) is present in the non-coding regions in *Po. spinulosa* upstream the 5’-end of the coding region, and a GC-rich motif (65 bp, 57% G and C) is present downstream the 3’-end of the coding region (Figure 3B). Additional to the full-length non-coding region sequences of the two minichromosomes, we also obtained partial sequences of the non-coding regions, 95–217 bp and 74–136 bp respectively, upstream and downstream the coding regions of the other nine minichromosomes of *Po. spinulosa*. As in *Po. asiatica*, two highly conserved sequence-motifs, 95 bp and 74 bp long respectively, are present in the sections of the non-coding regions upstream and downstream the coding regions of all of the 11 minichromosomes (see Additional file 4).

## Discussion

### Variation in the pattern of mt genome fragmentation between the two *Polyplax* rat lice

Only two of the 11 minichromosomes have identical gene content and gene arrangement between *Po. asiatica* and *Po. spinulosa*, revealing, for the first time, marked intra-genus variation in the pattern of mt genome fragmentation among blood-sucking lice (Figure 2). Comparison between the two *Polyplax* rat lice, and with the pig lice and the human lice, revealed the translocations of tRNA genes between minichromosomes in both *Po. asiatica* and *Po. spinulosa* after these two species split from their MRCA, and furthermore, the swap of identities between the two tRNA-leucine genes in *Po. spinulosa* after the split.

**Figure 4:**
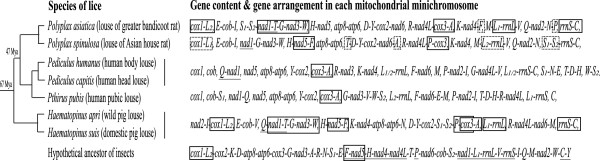
**Translocations of tRNA genes between mitochondrial minichromosomes and identity**-**swap between tRNA**-**leucine genes in the rat lice**, ***Polyplax asiatica *and *Polyplax spinulosa.*** Minichromosomes are separated by a comma (,). Genes on the same minichromosome are linked by a hyphen (-). Genes underlined have an opposite orientation of transcription relative to those not underlined. Minichromosomes and gene arrangements that are ancestral to the *Polyplax* rat lice, the human lice and the pig lice are indicated with solid-line boxes. tRNA genes that were translocated in the *Polyplax* lice are indicated with dot-line boxes. The two minichromosomes of *Po. asiatica* where the tRNA-leucine genes swapped identities are also indicated with dot-line boxes. The phylogeny and the date estimate for the common ancestors are after Light et al. [3].

**Figure 5:**
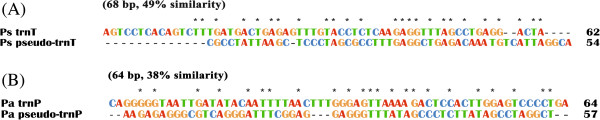
**Alignment of the nucleotide sequences between *trnT *and pseudo**-***trnT *of *Polyplax spinulosa *(A) and between *trnP *and pseudo-*trnP *of *Polyplax asiatica *(B).**

*RrnS*-*trnC* minichromosome is present in the rat louse, *Po. asiatica*, and the two *Haematopinus* species of the pig lice, indicating this minichromosome is ancestral to the rat lice, the pig lice and the human lice (Figure 4). In the rat louse, *Po. spinulosa*, however, *trnS*_*1*_-*trnS*_*2*_ has been inserted upstream *rrnS* gene (Figure 2B; Figure 4). The gene arrangement, ***nad1****-****trnT****-trnG-nad3-trnW* (genes in bold have opposite orientation of transcription to others), is present in *Po. asiatica* and the *Haematopinus* pig lice, indicating this arrangement to be ancestral to the rat lice, the pig lice and the human lice (Figure 4). In *Po. spinulosa*, however, *trnT* has been inserted upstream *trnD* gene, leaving a 54-bp pseudo-*trnT* between *nad1* and *trnG* (Figure 2B; Figure 5A). The gene arrangement, *cox3*-*trnA*, which is present in *Po. asiatica*, the *Haematopinus* pig lice and the human lice, is apparently ancestral to these lice. In *Po. spinulosa*, however, *trnA* has been inserted downstream *nad6* gene (Figure 2B; Figure 4).

Translocation of tRNA genes between minichromosomes also occurred in *Po. asiatica*. The gene arrangement, *trnP*-*cox3*, which is present in *Po. spinulosa* and the *Haematopinus* pig lice, is apparently ancestral to the rat lice, the pig lice and the human lice (Figure 4). In *Po. asiatica*, however, *trnP* has been inserted downstream *trnN*, leaving a 57-bp pseudo-*trnP* between *nad4L* and *cox3* (Figure 2A; Figure 5B). Unusually, there is a 189-bp noncoding region in between *trnN* and *trnP* (Figure 2A). The gene arrangement, *nad5*-*trnF*, which is ancestral to insects [12], is retained in *Po. spinulosa* and the *Haematopinus* pig lice. In *Po. asiatica*, however, *trnF* has been inserted downstream *nad4* gene (Figure 2A; Figure 4).

**Figure 6:**
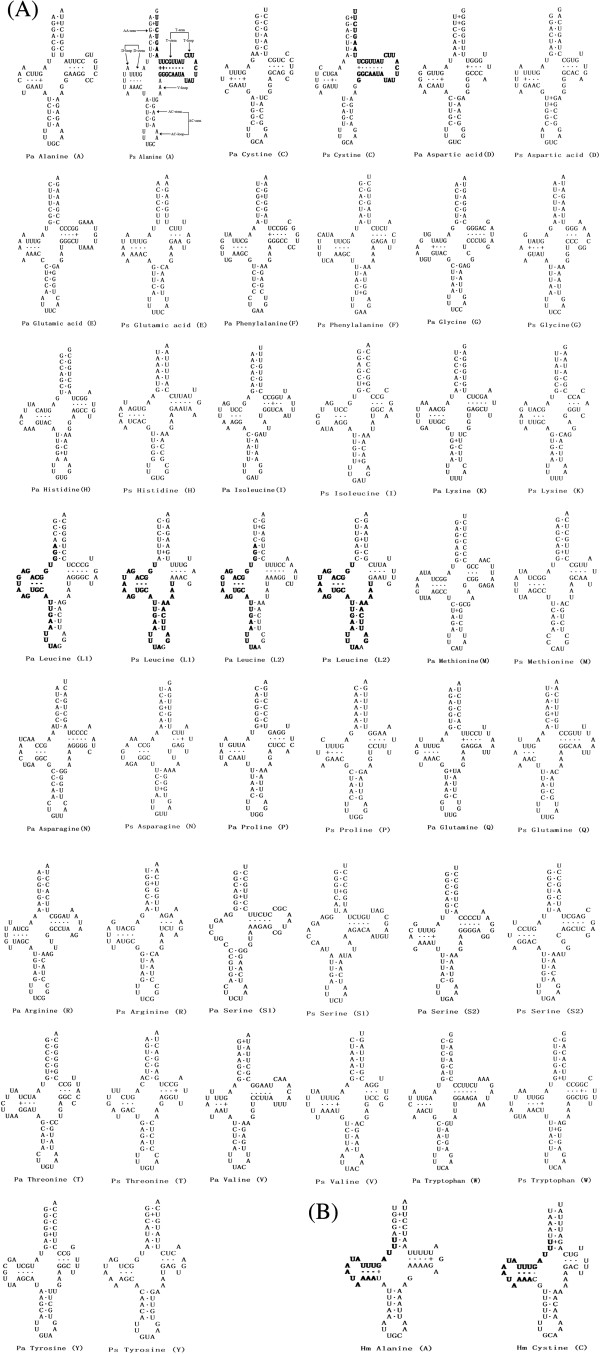
**Inferred secondary structures of the mitochondrial tRNAs of *Polyplax asiatica *(Pa) and *Polyplax spinulosa *(Ps) (A) and mitochondrial tRNAs for alanine and cysteine of the wallaby louse, *Heterodoxus macropus *(B).** Identical sequences shared between the two tRNAs for leucine are in bold, so are the identical sequences shared between tRNA-alanine and tRNA-cystine (see also Table 2). The 17-bp identical sequences shared between the two tRNAs of *Heterodoxus macropus* are in bold (see also Table 2).

The rat louse, *Po. asiatica*, and the *Haematopinus* pig lice retained an ancestral gene arrangement of insects, *cox1*-*trnL*_*2*_ (Figure 4) [12]. The gene arrangement, *trnL*_*1*_-*rrnL*, which is present in *Po. asiatica*, the *Haematopinus* pig lice, and the human head and body lice, is apparently ancestral to these lice. In *Po. spinulosa*, however, these two *trnL* genes swapped their positions: *trnL*_*1*_ lies downstream *cox1* in one minichromosome whereas *trnL*_*2*_ lies upstream *rrnL* in another minichromosome (Figure 2B). Given the high sequence similarity between *trnL*_*1*_ and *trnL*_*2*_ (Figure 6A; also see below), it is more likely that these two tRNA genes swapped their identities by recombination or point mutations at the third anti-codon positions than by translocations of these two genes between minichromosomes, as observed previously in the human head louse and body louse [11].

### Recombination between mt genes in parasitic lice

In the human lice and the pig lice, ten pairs of mt genes share stretches of identical sequences much longer than expected by chance, providing striking evidence for DNA recombination between mt genes and between minichromosomes [10-12]. As in the human lice and the pig lice, *trnL*_*1*_ and *trnL*_*2*_ in both rat lice, *Po. asiatica* and *Po. spinulosa*, share 28-bp and 25-bp identical sequences respectively (plus a 11-bp identical sequence immediately downstream with 1 bp in between in both species), which are 4–5 times longer than in the animals that have the typical mt genome organization (Table 2; Figure 6A). Recombination between *trnL*_*1*_ and *trnL*_*2*_ appears to be common in the blood-sucking lice and occurs likely more frequent than other mt genes.

**Table 2:** **The longest stretches of identical sequence shared by mitochondrial genes in two rat lice**, **three human lice**, **two pig lice**, **which have fragmented mitochondrial genomes**, **and six other species of bilateral animals that have the typical mitochondrial genomes**

**Pair of gene**		**The longest stretches of identical sequences shared**												
		**Rat lice**		**Human lice**			**Pig lice**		**Animals with typical mt genome organization**					
		***Pa***	***Ps***	***Pc***	***Ph***	***Pp***	***Has***	***Haa***	***Bm***	***Cb***	***Hm***	***Dy***	***Ce***	***Hos***
*trnL*_*1*_	*trnL*_*2*_	**28**	**25**, **11**	**33**, **32**	**33**, **32**	**35**, **32**	**16**, **10**, **9**	**16**, **10**, **9**	7	6	7	10	6	6
*cob*	*nad5*	12	**36**	12	12	13	13	13	12	16	14	13	13	12
*trnA*	*trnC*	6	**32**	6	6	6	6	6	NA	7	**17**	7	8	6
*nad4*	*nad5*	12	18	**127**, **30**	**127**, **30**	NA	12	12	13	15	15	16	14	11
*nad5*	*rrnL*	13	13	**99**	**99**	10	11	10	12	14	13	15	16	10
*trnG*	*trnR*	5	5	**28**, **14**	**28**, **14**	**32**, **26**	5	5	5	6	7	6	8	6
*cox1*	*nad4L*	9	11	10	10	**29**	11	11	13	11	14	13	12	10
*nad2*	*rrnL*	10	11	**26**	**26**	10	10	11	13	11	14	13	12	10
*trnP*	*trnT*	6	7	7	7	NA	**26**	**26**	6	8	8	9	10	7
*atp8*	*trnG*	7	9	**26**	**26**	9	6	6	10	11	11	12	NA	6
*atp8*	*nad2*	12	10	10	10	8	**25**	**25**	10	14	12	14	NA	11
*trnI*	*trnT*	10	8	6	6	**16**	6	6	6	5	7	7	9	6

Note: Abbreviations of species names are: *Pa*, *Polyplax asiatica* (louse of the greater bandicoot rat); *Ps*, *Polyplax spinulosa* (louse of the Asian house rat); *Pc*, *Pediculus capititis* (human head louse); *Ph*, (human body louse); *Pp*, *Pthirus pubis* (*human pubic louse*); *Has*, *Haematopinus suis* (domestic pig louse); *Haa*, *Haematopinus apri* (wild pig louse); *Bm*, *Bothriometopus macrocnemis* (screamer louse); *Cb*, *Campanulotes bidentatus* (pigeon louse); *Hm*, *Heterodoxus macropus* (wallaby louse); *Dy*, *Drosophila yakuba* (fruitfly); *Ce*, *Caenorhabditis elegans* (roundworm); *Hos*, *Homo sapiens* (human); *NA*, not available. Stretches of shared identical sequences longer than expected by chance are in bold.

Two other pairs of mt genes in the rat louse, *Po. spinulosa*, also share identical sequences, 36 bp between *cob* and *nad5*, and 32 bp between *trnA* and *trnC*, which are 3–5 times longer than in other animals (Table 2). Recombination between *cob* and *nad5*, and between *trnA* and *trnC* in *Po. spinulosa*, appears to be recent evolutionary events and less common as these two pairs of genes do not share longer-than-expected sequences in the other rat louse, *Po. asiatica*, nor in the human lice and the pig lice (Table 2). Intriguingly, *trnA* and *trnC* of the wallaby louse, *Heterodoxus macropus*, share 17 bp identical sequence (Figure 6B; Table 2), which is 2–3 times longer than expected by chance, indicating that recombination between mt genes may also occur in the animals that have the typical mt genome organization although it is much rarer than in the blood-sucking lice, which have fragmented mt genomes.

It was suggested that recombination between tRNA genes could affect the secondary structure of their corresponding tRNAs [11]. This is, indeed, the case for tRNA-Alanine and tRNA-Cystine in the rat lice. The 32-bp identical sequence shared between *trnA* and *trnC* in *P. spinulosa* falls on the AA-arm and T-arm of tRNA-Alanine and tRNA-Cystine. These two tRNAs have the longest T-arms among all of the tRNAs of *Po. spinulosa*, with 8 pairs and 7 pairs respectively at the T-stems and 9 nt at the T-loop, in comparison to the average 4.2 pairs and 4.6 nt of the 22 tRNAs (Table 3; Figure 6A). In contrast, in the other rat louse, *Po. asiatica*, in which *trnA* and *trnC* do not share longer-than-expected sequences, the T-arms of tRNA-Alanine and tRNA-Cystine have 5 pairs and 4 pairs respectively at the T-stems, and 7 nt and 5 nt at the T-loop, which are very close to the average 4.6 pairs and 5.9 nt of the 22 tRNAs (Table 3).

**Table 3:** **Length of T**-**stem**, **T**-**loop and T**-**arm of the mitochondrial tRNAs of the rat lice**, ***Polyplax asiatica *(*Pa*) and *Polyplax spinulosa *(*Ps*)**

**tRNA**	**Anti-codon**	***Pa*T-stem(pairs)**	***Ps*T-stem(pairs)**	***Pa*T-loop(nt)**	***Ps*T-loop(nt)**	***Pa*T-arm(nt)**	***Ps*T-arm(nt)**
Alanine (A)	UGC	5	8	7	9	17	25
Cystine (C)	GCA	4	7	5	9	13	23
Aspartic acid (D)	GUC	4	4	3	4	11	12
Glutamic acid (E)	UUC	5	3	11	4	21	10
Phenylalanine (F)	GAA	5	4	6	4	16	12
Glycine (G)	UCC	5	3	5	6	15	12
Histidine (H)	GUG	4	5	4	3	12	13
Isoleucine (I)	GAU	5	3	6	4	16	10
Lysine (K)	UUU	5	3	3	3	13	9
Leucine (L_1_)	UAG	5	4	4	3	14	11
Leucine (L_2_)	UAA	5	4	6	4	16	12
Methionine (M)	CAU	3	4	10	3	16	11
Asparagine (N)	GUU	5	3	4	3	14	9
Proline (P)	UGG	4	4	4	3	12	11
Glutamine (Q)	UUG	5	5	6	6	16	16
Arginine (R)	UCG	5	3	6	4	16	10
Serine (S_1_)	UCU	5	5	8	10	18	20
Serine (S_2_)	UGA	5	5	6	4	16	14
Threonine (T)	UGU	3	4	5	3	11	11
Valine (V)	UAC	5	3	9	4	19	10
Tryptophan (W)	UCA	6	5	8	4	20	14
Tyrosine (Y)	GUA	3	3	4	4	10	10
Total		101	92	130	101	332	285
Mean		4.6	4.2	5.9	4.6	15.1	13.0

### A possible link between the extent of mt genome fragmentation and the length of life cycle

The rat lice in the genus *Polyplax* share their MRCA with the human lice ~47 Mya, and share their MRCA with the pig lice ~67 Mya (Figure 4) [3]. The mt genomes of the two *Polyplax* species we sequenced in the present study have 3.4 genes on average on each minichromosome and thus, are less fragmented than those of the human lice (2.1 and 2.4 genes per minichromosome) but are more fragmented than those of the pig lice (4.1 genes per minichromosome). Clearly, the extent of mitochondrial genome fragmentation varies among different lineages of the blood-sucking lice.

The pig lice, *Haematopinus suis* and *Haematopinus apri*, have the least fragmented mt genomes among the blood-sucking lice whose mt genomes have been sequenced completely or near completely sequenced to date [12]. Intriguingly, these two *Haematopinus* species have the largest body size (up to 6 mm long) among the blood-sucking lice known [30]. The body size of the blood-sucking lice, however, does not appear to be linked to the extent of mt genome fragmentation as the human lice, *Pediculus humanus* and *Pediculus capitis*, have larger body size (up to 3.6 mm long) than the *Polyplax* rat lice (up to 1.5 mm long) but have more fragmented mt genomes than the *Polyplax* rat lice (Table 4).

**Table 4:** **Body length**, **life**-**cycle length and number of mitochondrial genes per minichromosome of five species of blood**-**sucking lice**

**Species of lice**	**Pig louse*Haematopinus suis***	**Rat louse*Polyplax spinulosa***	**Human pubic louse*Pthirus pubis***	**Human body louse*Pediculus humanus***	**Human head louse*Pediculus capitis***
Adult body length (mm)	4-6	0.6-1.5	1.3-2.0	2.3-3.6	2.1-3.3
Length of life cycle (days)	29-48	25-28	16-25	14-20	13-17
Mitochondrial genes per minichromosome	4.1	3.4	2.4	2.1	2.1

Note: The information of body length and life cycle was from Dynum et al. [31], Florence [32], Wall and Shearer [30] and Baker et al. [33] for the pig louse; from Baker [22] and Goodwin et al. [34] for the rat louse; from Buxton [35] and Service [36] for the human pubic louse; and from Buxton [35], Buxton [37] and Service [36] for the human body louse and head louse.

Instead, the length of life cycle of the blood-sucking lice appears to be linked to the extent of mt genome fragmentation. The pig louse, *H. suis*, has the longest life cycle (29–48 days depending on weather; no data available to *H. apri*) and the least fragmented mt genome among the blood-sucking lice whose mt genomes have been sequenced completely or near completely to date (Table 4). In contrast, the human head louse, *Pe. capitis*, and the body louse, *Pe. humanus*, have the shortest life cycle (13–20 days) and the most fragmented mt genomes. The rat louse, *Po. spinulosa*, has both the length of life cycle (25–28 days; no data available to *Po. asiatica*) and the extent of mt genome fragmentation in between the pig lice and the human head and body lice. Furthermore, the human pubic louse, *Pt. pubis*, has both the length of life cycle (16–25 days) and the extent of mt genome fragmentation in between the rat lice and the human head and body lice. The inverse link between the length of life cycle and the extent of mt genome fragmentation, indicated in the present study by limited number of species, can be tested further with data from more species of blood-sucking lice, and other eukaryotes that have fragmented mt genomes such as the ichthyosporean protists [16], diplonemid protists [17] and box jellyfish [18]. Currently, there is no sufficient data and information, to our knowledge, to link the length of life cycle (or other life-history factors) with the extent of mt genome fragmentation in these eukaryotes. Theoretically, if mitochondrial genome fragmentation was a continuous process and each generation contributed approximately equally towards fragmentation, then it would be expected that the extent of fragmentation be inversely linked to the length of life cycle.

## Conclusions

We sequenced the mt genomes of *Po. asiatica* and *Po. spinulosa*, collected from the greater bandicoot rat, *B. indica*, and the Asian house rat, *R. tanezumi*. We identified all of the 37 mt genes common to animals in these rat lice. The mt genes of *Po. asiatica* and *Po. spinulosa* are on 11 minichromosomes; each minichromosome is 2–4 kb long and has 2–7 genes. The two rat lice share the same pattern for the distribution of the protein-coding genes and rRNA genes over the minichromosomes, but differ in the pattern for the distribution of 8 of the 22 tRNA genes. The mt genomes of the two *Polyplax* rat lice have 3.4 genes, on average, on each minichromosome and thus, are less fragmented than those of the human lice (2.1 and 2.4 genes per minichromosome) but are more fragmented than those of the pig lice (4.1 genes per minichromosome). Our results revealed distinct patterns of mt genome fragmentation between the two *Polyplax* species of rat lice. We also showed that the extent of mt genome fragmentation appears to have an inverse link with the length of life cycle of the rat lice, the human lice and the pig lice. Whether, or not, the extent of mt genome fragmentation is indeed linked to the length of life cycle, however, needs to be tested further with data from more species and broader phylogenetic ranges of blood-sucking lice, and other eukaryotes that have fragmented mt genomes.

### Availability of supporting data

The nucleotide sequences of the mt genomes of the rat lice supporting the results of this article have been deposited in GenBank [accession numbers KF647751–KF647772; http://www.ncbi.nlm.nih.gov/].

## Abbreviations

μl: Microliter; atp6 and atp8: Genes for ATP synthase subunits 6 and 8; bp: Base pair; cob: Gene for cytochrome b; cox1 cox2 and cox3: Genes for cytochrome *c* oxidase subunits 1, 2 and 3; DNA: Deoxyribonucleic acid; kb: Kilo base pair; min: Minute; MRCA: Most recent common ancestor; Mt: Mitochondrial; Mya: Million years ago; nad1, nad2, nad3, nad4, nad4L, nad5 and nad6: Mitochondrial genes for NADH dehydrogenase subunits 1–6 and *4 L*; PCR: Polymerase chain reaction; RNA: Ribonucleic acid; rRNA: Ribosomal RNA; rrnS and rrnL: Genes for small and large subunits of ribosomal RNA; sec: Second; T: Thymine; tRNA: transfer RNA; tRNA: transfer RNA; trnA or A: tRNA gene for alanine; trnC or C: tRNA gene for cysteine; trnD or D: tRNA gene for aspartic acid; trnE or E: tRNA gene for glutamic acid; trnF or F: tRNA gene for phenylalanine; trnG or G: tRNA gene for glycine; trnH or H: tRNA gene for histidine; trnI or I: tRNA gene for isoleucine; trnK or K: tRNA gene for lysine; trnL1 or L1: tRNA gene for leucine (anticodon NAG); trnL2 or L2: tRNA gene for leucine (anticodon YAA); trnM or M: tRNA gene for methionine; trnN or N: tRNA gene for asparagine; trnP or P: tRNA gene for proline; trnQ or Q: tRNA gene for glutamine; trnR or R: tRNA gene for arginine; trnS1 or S1: tRNA gene for serine (anticodon NCU); trnS2 or S2: tRNA gene for serine (anticodon NGA); trnT or T: tRNA gene for threonine; trnV or V: tRNA gene for valine; trnW or W: tRNA gene for tryptophan; trnY or Y: tRNA gene for tyrosine; U: Uracil.

## Competing interests

The authors declare that they have no competing interests.

## Authors’ contributions

WGD, SS, DCJ, XGG and RS designed the research. WGD, SS and RS performed the research. DCJ, XGG and RS contributed reagents and materials. WGD, SS and RS analyzed the data. WGD and RS wrote the manuscript. All authors have read and approved the final manuscript.

## Supplementary Material

Additional file 1 **PCR primers used to amplify and sequence the mitochondrial genomes of the rat lice, *Polyplax asiatica *(*Pa*) and *Polyplax spinulosa *(*Ps*).** Click here for file

Additional file 2 **PCR primers used to verify the mitochondrial minichromosomes of the rat lice, *Polyplax asiatica *and *Polyplax spinulosa.***
Click here for file

Additional file 3 **Alignment of nucleotide sequences of parts of the non-coding regions upstream (A) and downstream (B) of the coding regions of the 11 mitochondrial minichromosomes of *Polyplax asiatica, *the louse of the greater bandicoot rat, *Bandicota indica.*** 57F and 57R are the primers used to amplify the coding regions of all mitochondrial minichromosomes of *Polyplax asiatica*. Click here for file

Additional file 4 **Alignment of nucleotide sequences of parts of the non-coding regions upstream (A) and downstream (B) of the coding regions of the 11 mitochondrial minichromosomes of *Polyplax spinulosa *, the louse of the Asian house rat, *Rattus tanezumi*.** 301F and 301R are the primers used to amplify the coding regions of all mitochondrial minichromosomes of *Polyplax spinulosa*. Click here for file

## References

[B1] Kim KC Kim KC Evolution and host associations of Anoplura Coevolution of Parasitic Arthropods and Mammals 1985 New York: John Wiley and Sons Press 197 231

[B2] Lehane MJ The Biology of the Blood Sucking Insects 2005 2 New York: Cambridge University Press

[B3] Light JE Smith VS Allen JM Durden LA Reed RL Evolutionary history of mammalian sucking lice (Phthiratpera: Anoplura) BMC Evol Biol 2010 15 292 10.1186/1471-2148-10-292 20860811 PMC2949877

[B4] Kim KC Ludwig HW The family classification of the Anoplura Syst Entomol 1978 15 249 284 10.1111/j.1365-3113.1978.tb00120.x

[B5] Kim KC Service MW Evolutionary parallelism in Anoplura and eutherian mammals Biosystematics of Haematophagous Insects 1988 37 Oxford: Oxford University Press 91 114

[B6] Durden LA Musser GG The sucking lice (Insecta, Anoplura) of the world: a taxonomic checklist with records of mammalian hosts and geographical distributions Bull Amer Mus Nat Hist 1994 15 1 90

[B7] Durden LA Musser GG The mammalian hosts of the sucking lice (Anoplura) of the world: a host-parasite list Bull Soc Vector Ecol 1994 15 130 168

[B8] Boore JL Animal mitochondrial genomes Nucleic Acids Res 1999 15 1767 1780 10.1093/nar/27.8.1767 10101183 PMC148383

[B9] Lavrov DV Key transitions in animal evolution: a mitochondrial DNA perspective Integr Comp Biol 2007 15 734 743 10.1093/icb/icm045 21669754

[B10] Shao R Kirkness EF Barker SC The single mitochondrial chromosome typical of animals has evolved into 18 minichromosomes in the human body louse, *Pediculus humanus* Genome Res 2009 15 904 912 10.1101/gr.083188.108 19336451 PMC2675979

[B11] Shao R Zhu XQ Barker SC Herd K Evolution of extensively fragmented mitochondrial genomes in the lice of humans Genome Biol Evol 2012 15 1088 1101 10.1093/gbe/evs088 23042553 PMC3514963

[B12] Jiang HW Barker SC Shao R Substantial variation in the extent of mitochondrial genome fragmentation among blood-sucking lice of mammals Genome Biol Evol 2013 15 1298 1308 10.1093/gbe/evt094 23781098 PMC3730346

[B13] Suga K Welch DBM Tanaka Y Sakakura Y Hagiwarak A Two circular chromosomes of unequal copy number make up the mitochondrial genome of the rotifer *Brachionus plicatilis* Mol Biol Evol 2008 15 1129 1137 10.1093/molbev/msn058 18326862

[B14] Wei DD Shao R Yuan ML Dou W Barker SC Wang JJ The multipartite mitochondrial genome of *Liposcelis bostrychoophila*: insights into the evolution of mitochondrial genomes in bilateral animals PLoS One 2012 15 e33973 10.1371/journal.pone.0033973 22479490 PMC3316519

[B15] Gibson T Blok VC Dowton M Sequence and characterization of six mitochondrial subgenomes from *Globodera rostochiensis*: Multipartite structure is conserved among close nematode relatives J Mol Evol 2007 15 308 315 10.1007/s00239-007-9007-y 17674076

[B16] Burger G Forget L Zhu Y Gray MW Lang BF Unique mitochondrial genome architecture in unicellular relatives of animals Proc Natl Acad Sci U S A 2003 15 892 897 10.1073/pnas.0336115100 12552117 PMC298697

[B17] Marande W Lukes J Burger G Unique mitochondrial genome structure in diplonemids, the sister group of kinetoplastids Eukaryot Cell 2005 15 1137 1146 10.1128/EC.4.6.1137-1146.2005 15947205 PMC1151984

[B18] Smith DR Kayal E Yanagihara AA Collins AG Pirro S Keeling PJ First complete mitochondrial genome sequence from a *box jellyfish* reveals a highly fragmented linear architecture and insights into telomere evolution Genome Biol Evol 2012 15 52 58 10.1093/gbe/evr127 22117085 PMC3268669

[B19] Chin TH Taxonomy and fauna of sucking lice (Anoplura) in China 1999 Beijing: Science Press 1 132

[B20] Crystal MM The mechanism of transmission of Haemobartonella muris (Mayer) of rats by the spined rat louse, *Polyplax spinulosa* (Burmeister) J Parasitol 1958 15 603 606 10.2307/3274543 13621318

[B21] Neimark H Johansson KE Rikihisa Y Tully JG Revision of haemotrophic *Mycoplasma* species names Int J Syst Evol Microbiol 2002 15 683 11931184 10.1099/00207713-52-2-683

[B22] Baker DG Suckow MA, Weisbroth SH, Franklin CL Parasitic diseases The Laboratory Rat 2006 Second Elsevier Press A Volume in American College of Laboratory Animal Medicine. 453 478

[B23] Kearse M Moir R Wilson A (11 co-authors): Geneious basic: An integrated and extendable desktop software platform for the organization and analysis of sequence data Bioinformatics 2012 15 1647 1649 10.1093/bioinformatics/bts199 22543367 PMC3371832

[B24] Lowe TM Eddy SR tRNAscan-SE: a program for improved detection of transfer RNA genes in genomic sequence Nucleic Acids Res 1997 15 955 964 9023104 10.1093/nar/25.5.955 PMC146525

[B25] Laslett D Canback B ARWEN: a program to detect tRNA genes in metazoan mitochondrial nucleotide sequences Bioinformatics 2008 15 172 175 10.1093/bioinformatics/btm573 18033792

[B26] Altschul SF Madden TL Schaffer AA Zhang J Zhang Z Miller W Lipman DJ Gapped BLAST and PSI-BLAST: a new generation of protein database search programs Nucleic Acids Res 1997 15 3389 3402 10.1093/nar/25.17.3389 9254694 PMC146917

[B27] Gish W States DJ Identification of protein coding regions by database similarity search Nature Genet 1993 15 266 272 10.1038/ng0393-266 8485583

[B28] Rice P Longden I Bleasby A EMBOSS: The European molecular biology open software suite Trends Genet 2000 15 276 277 10.1016/S0168-9525(00)02024-2 10827456

[B29] Larkin MA Clustal W and Clustal X version 2.0 Bioinformatics 2007 15 2947 2948 10.1093/bioinformatics/btm404 17846036

[B30] Wall R Shearer D Veterinary Ectoparasites: Biology, Pathology and Control 2001 Second Wiley-Blackwell Press

[B31] Dynum E Ward C Meeks D Hog louse, *Haematopinus suis*, population growth and distribution on its host Southwest Entomol 1978 15 106 112

[B32] Florence L The hog louse, *Haematopinus suis* Linné: its biology, anatomy and histology Cornell University Agric Exp Station Mem 1921 15 635 743

[B33] Baker JR Apperson CS Arends JJ Insect and related pests of man and animals The North Carolina Agric Ext Serv 2013 http://ipm.ncsu.edu/AG369/

[B34] Goodin BS Yarbrough LW Head KL Rats and Mice: Parasitic Diseases 2000 University of Washington Health Sciences Center for Educational Resources and the American College of Laboratory Animal Medicine

[B35] Buxton PA The louse, an account of the lice which infest man, their medical importance and control 1947 2 London: Edward Arnold Press

[B36] Service M Medical Entomology for Students 2012 5 New York: Cambridge University Press

[B37] Buxton PA The louse: Present knowledge and future work Trans R Soc Trop Med Hyg 1940 15 365 379 10.1016/S0035-9203(40)90039-6

