# Supplementary material for: Fragmented mitochondrial genomes of the rat lice, Polyplax asiatica and Polyplax spinulosa: intra-genus variation in fragmentation pattern and a possible link between the extent of fragmentation and the length of life cycle
Source: BMC Genomics. 2014 Jan 18;15:44. doi: 10.1186/1471-2164-15-44 (PMC3901344; doi:10.1186/1471-2164-15-44)
Supplement: Additional file 1 — PCR primers used to amplify and sequence the mitochondrial genomes of the rat lice, Polyplax asiatica (Pa) and Polyplax spinulosa (Ps). [file 1471-2164-15-44-S1.pdf]

Additional file 1 - PCR primers used to amplify and sequence the mitochondrial genomes of the rat lice, *Polyplax asiatica* (*Pa*) and *Polyplax spinulosa* (*Ps*)

Primer	Target gene	Sequence (5' to 3')	Species
mtd6	*cox1*	GGAGGATTTGGAAATTGATTAGTTCC	*Pa*
mtd9	*cox1*	CCCGGTAAAATTAAAATATAAACTTC	*Pa*
16SF	*rrnL*	TTAATTCAACATCGAGGTCGCAA	*Pa*
Lx16SR	*rrnL*	GACTGTGCTAAGGTAGCATAAT	*Pa*
cox57F	*cox57*	CAGCGTTTCTCTTGTTACTTTCGCTCCCAG	*Pa*
cox57R	*cox57*	GCCAAATCCACTGAAGCTCCGGGCTGACC	*Pa*
16S57F	*16S57*	CGAGAAGACCCTGTAGAGCTTTAATCTGGC	*Pa*
16S57R	*16S57*	CCCTCTATCCTATCTGGGAGTTTTCACCC	*Pa*
57F	*M57*	ACCTGGCCCGTGGGGGAGTCAAATCTT	*Pa*
57R	*M57*	TGGAAGCAGGAGGCTCTGGAAGATGAC	*Pa*
12SA	*rrnS*	TACTATGTTACGACTTAT	*Ps*
12SB	*rrnS*	AAACTAGGATTAGATACCC	*Ps*
16SF	*rrnL*	TTAATTCAACATCGAGGTCGCAA	*Ps*
Lx16SR	*rrnL*	GACTGTGCTAAGGTAGCATAAT	*Ps*
12S301F	*12S301*	CATATGCCTCTGAATAGACTCTCTACCGCCA	*Ps*
12S301R	*12S301*	CAGTAAACGAAACTGCCCGAACACTTTAC	*Ps*
16S301F	*16S301*	GAAGAGACAGTTGAACCTCGGTTTACCC	*Ps*
16S301R	*16S301*	GGCTGAGAGTTACCTGGGAGG GGGCTATAC	*Ps*
301F	*M301*	GCAGAAGGCACTTTAGGGCTTAAGTCCCTG	*Ps*
301R	*M301*	CTCAACCTTTGAGGGGTTGTTTAGACGTG	*Ps*
