# Supplementary material for: Fragmented mitochondrial genomes of the rat lice, Polyplax asiatica and Polyplax spinulosa: intra-genus variation in fragmentation pattern and a possible link between the extent of fragmentation and the length of life cycle
Source: BMC Genomics. 2014 Jan 18;15:44. doi: 10.1186/1471-2164-15-44 (PMC3901344; doi:10.1186/1471-2164-15-44)
Supplement: Additional file 2 — PCR primers used to verify the mitochondrial minichromosomes of the rat lice, Polyplax asiatica and Polyplax spinulosa. [file 1471-2164-15-44-S2.pdf]

Additional file 2 - PCR primers used to verify the mitochondrial minichromosomes of the rat lice, *Polyplax asiatica* and *Polyplax spinulosa*

Primer	Target gene	Sequence (5' to 3')
57M1F	*atp6*	CTAATGGGGGTTTACATGGTGCTCCTAAC
57M1R	*atp6*	CTCAAAGCTAAAGGCTTACCTTCCTTCAGC
57M2F	*cob*	GATTTCTTAGGGGTGTCCGTTTTAGGGAGG
57M2R	*cob*	GAGATGAAATAAGGGTGAAAGGCCACTTTG
57M3F	*cox1*	GGGAGTTTAGGGATAATTTATGCCATGAGG
57M3R	*cox1*	CTTCCCCATTTCATCGGAAATTATGTGAG
57M4F	*cox2*	GACTCGTACCTTTTGCCTAAGGAGTGGGAG
57M4R	*cox2*	CAAGAAGGTCTCTAGGCTCGTAGGAGAG
57M5F	*trnR*	CAAGTTGGCCTAAGATAATAGGCTTCACC
57M5R	*trnR*	GGCCGAAACTACATGCTAACAAATAGC
57M6F	$trnS_1$	GGTAAAGAGCGTAACGCCTCTTCCTTGCC
57M6R	$trnS_1$	CCTTGATTAGAAGTCACGGGCAGACTTCC
57M7F	*nad2*	GGTCTCAGTTTCCTTGGACTAACTCAGC
57M7R	*nad2*	GAAGAAATTAAATCCCTCTCTGCCCTAGCC
57M8F	*nad4*	GGAAGCATTATCCTGGCCGGATACCTTC
57M8R	*nad4*	CCTAAGGGGAGCCTCCACATGAGCCTTTG
57M9F	*nad5*	CATTCCTCCACCTTGGTTACTGCTGGTC
57M9R	*nad5*	CTAAACTTCTAACTGGGGTAGGTGCCGC
57M10F	*rrnS*	CCTTCCCTGTTATGTTTTGTACACCGCTG
57M10R	*rrnS*	GATATTCGGGCGGCTTCGATTAGGGACATG
57M11F	*rrnL*	CCTGTAGAGCTTTAATCTGGCTAAACCAG
57M11R	*rrnL*	GTCTTCTCGTCCCTCTATCCTATCTGGGAG

301M1F	*atp6*	GGGAGGGGCTATCTACATGTTCTCAAAG
301M1R	*atp6*	CATATGGGGAGGCAAATTGTCAATCTCC
301M2F	*cob*	GGGCTAAGTCCTCATTCCCATAAAGTAGC
301M2R	*cob*	GGGGTTTGATCTTCCCTTTTCGTGGAG
301M3F	*cox1*	GGCGAGCTTATTTTACTAGGGCAACGATG
301M3R	*cox1*	CATCTAGCCCAACAGTGAATATGTGGTGCG
301M4F	*cox2*	CTGGTCATACGAAGAGTATAAGCCTCAGC
301M4R	*cox2*	GGCCAATTACCTTGGATATAATCGCTGG
301M5F	*trnR*	CGGCCTAACAGAATCTAGAAAGATTGGTC
301M5R	*trnR*	CCGCATAAATCATGCTCTTGCCCATAAGC
301M6F	*trnG*	CCAGTTAAAAGGTCCCGGCAAAGGGTTAAG
301M6R	*trnG*	CTTAAGATTTCGCCTATTAAGCTCCCTAGC
301M7F	*nad2*	GATCGAGGAGTCAATTCGACGGGCTATAG
301M7R	*nad2*	CTCCCTTGATTGAAACAAGAGTCGAGAGG
301M8F	*nad4*	GCATCTACCTCTGGTAGAGTGTTGTTAGCG
301M8R	*nad4*	CAACATGCGCCTTTGGCAGCCAGTAATGA
301M9F	*nad5*	GAGGACGACAAGACTCTCGGTCTCTTATTC
301M9R	*nad5*	CTCCTCATGGAGAATAACTCCTGCAACC
301M10F	*rrnS*	CAGTAAACGAAACTGCCCGAACACTTTAC
301M10R	*rrnS*	CATATGCCTCTGAATAGACTCTCTACCGCC
301M11F	*rrnL*	GAAGGCTAGAATGAAAGGGTAAACCGAGG
301M11R	*rrnL*	GGCAAATTATTATGCTACCTTAGCACGGTC

Note: Primers with “57” are for *Polyplax asiatica*; those with “301” are for *Polyplax spinulosa*.
