# Supplementary material for: Fragmented mitochondrial genomes of the rat lice, Polyplax asiatica and Polyplax spinulosa: intra-genus variation in fragmentation pattern and a possible link between the extent of fragmentation and the length of life cycle
Source: BMC Genomics. 2014 Jan 18;15:44. doi: 10.1186/1471-2164-15-44 (PMC3901344; doi:10.1186/1471-2164-15-44)
Supplement: Additional file 3 — Alignment of nucleotide sequences of parts of the non-coding regions upstream (A) and downstream (B) of the coding regions of the 11 mitochondrial minichromosomes of Polyplax asiatica, the louse of the greater bandicoot rat, Bandicota indica. 57F and 57R are the primers used to amplify the coding regions of all mitochondrial minichromosomes of Polyplax asiatica. [file 1471-2164-15-44-S3.pdf]

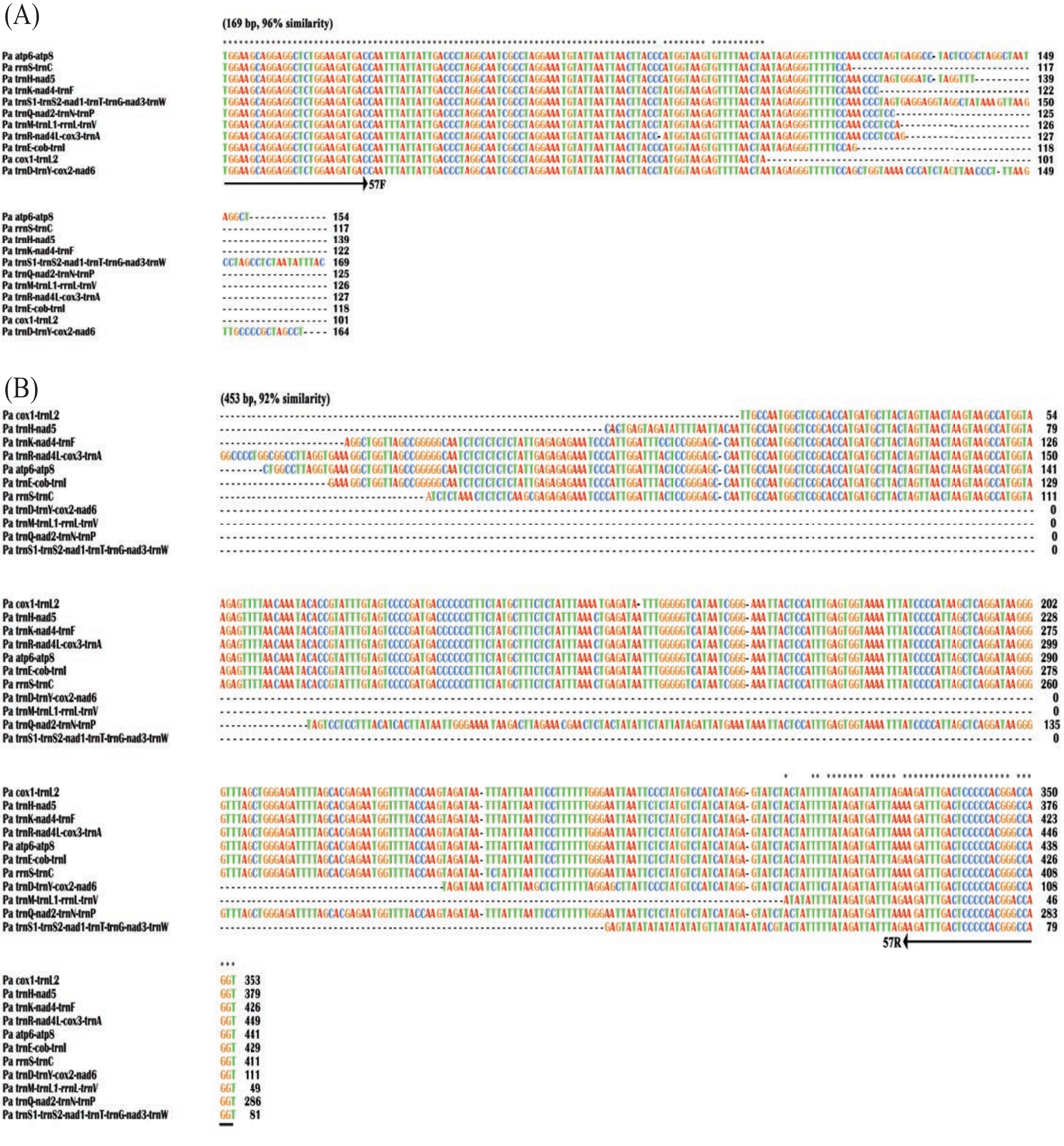

Additional file 3 - Alignment of nucleotide sequences of parts of the non-coding regions upstream (A) and downstream (B) of the coding regions of the 11 mitochondrial minichromosomes of *Polyplax asiatica*, the louse of the greater bandicoot rat, *Bandicota indica*. 57F and 57R are the primers used to amplify the coding regions of all mitochondrial minichromosomes of *Polyplax asiatica.*
