# Supplementary material for: Fragmented mitochondrial genomes of the rat lice, Polyplax asiatica and Polyplax spinulosa: intra-genus variation in fragmentation pattern and a possible link between the extent of fragmentation and the length of life cycle
Source: BMC Genomics. 2014 Jan 18;15:44. doi: 10.1186/1471-2164-15-44 (PMC3901344; doi:10.1186/1471-2164-15-44)
Supplement: Additional file 4 — Alignment of nucleotide sequences of parts of the non-coding regions upstream (A) and downstream (B) of the coding regions of the 11 mitochondrial minichromosomes of Polyplax spinulosa , the louse of the Asian house rat, Rattus tanezumi. 301F and 301R are the primers used to amplify the coding regions of all mitochondrial minichromosomes of Polyplax spinulosa. [file 1471-2164-15-44-S4.pdf]

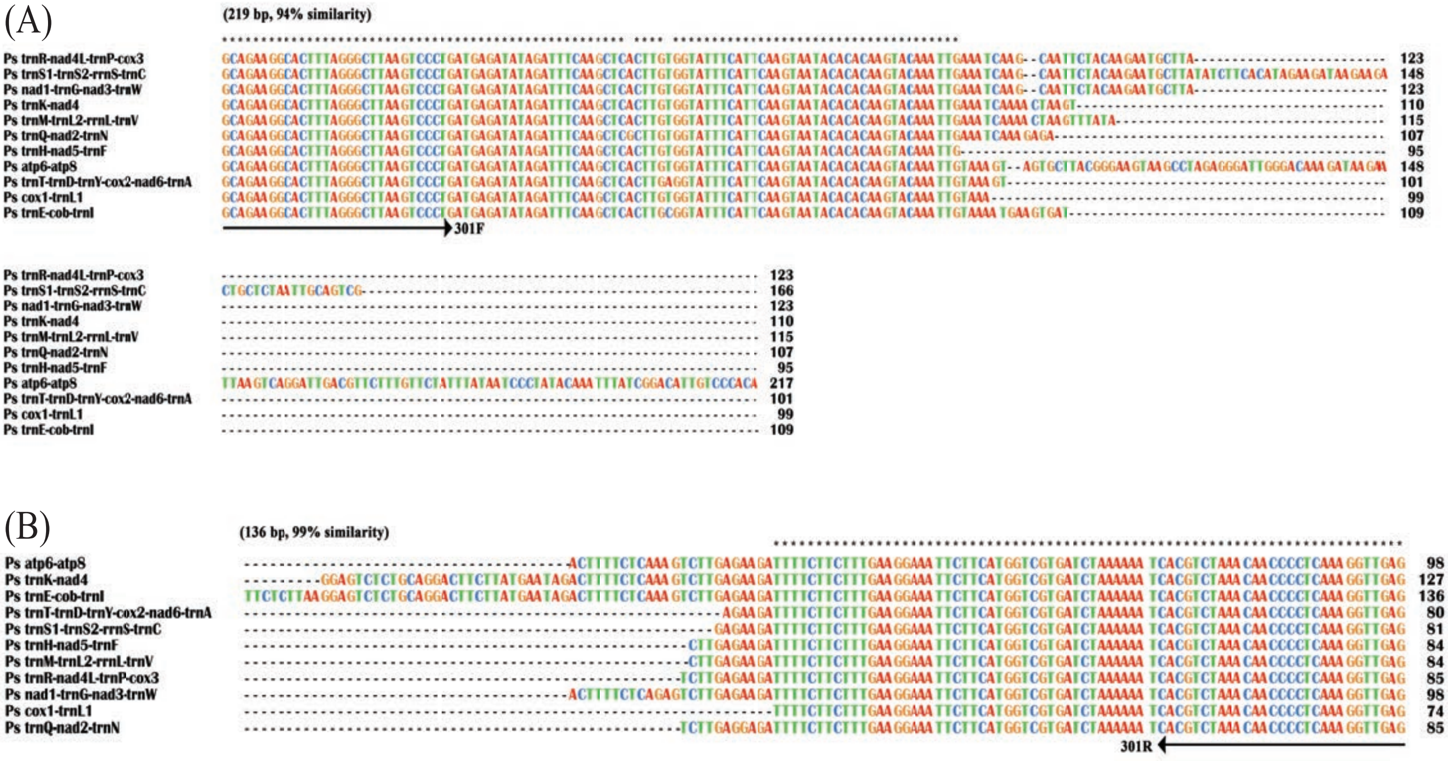

Additional file 4 - Alignment of nucleotide sequences of parts of the non-coding regions upstream (A) and downstream (B) of the coding regions of the 11 mitochondrial minichromosomes of *Polyplax spinulosa*, the louse of the Asian house rat, *Rattus tanezumi*. 301F and 301R are the primers used to amplify the coding regions of all mitochondrial minichromosomes of *Polyplax spinulosa.*
